# Synthesis of Dolichols in *Candida albicans* Is Co-Regulated with Elongation of Fatty Acids

**DOI:** 10.3390/ijms23010409

**Published:** 2021-12-30

**Authors:** Anna Janik, Urszula Perlińska-Lenart, Katarzyna Gawarecka, Justyna Augustyniak, Ewelina Bratek-Gerej, Przemysław Bernat, Sebastian Piłsyk, Patrycja Skalmowska, Grażyna Palamarczyk, Ewa Swiezewska, Joanna S. Kruszewska

**Affiliations:** 1 Institute of Biochemistry and Biophysics, Polish Academy of Sciences, Pawińskiego 5a, 02-106 Warsaw, Poland; annaj@ibb.waw.pl (A.J.); lenart@ibb.waw.pl (U.P.-L.); katag@ibb.waw.pl (K.G.); ewelina.bratek1@wp.pl (E.B.-G.); seba@ibb.waw.pl (S.P.); eszeweria@vp.pl (P.S.); gp@ibb.waw.pl (G.P.); ewas@ibb.waw.pl (E.S.); 2 Mossakowski Medical Research Centre, Polish Academy of Sciences, Pawińskiego 5, 02-106 Warsaw, Poland; jaugustyniak@imdik.pan.pl; 3 Faculty of Biology and Environmental Protection, University of Lodz, Banacha 12/16, 90-237 Lodz, Poland; przemyslaw.bernat@biol.uni.lodz.pl

**Keywords:** *Candida albicans*, Tsc13 *trans*-2 enoyl-CoA reductase, Dfg10 polyprenyl reductase

## Abstract

Protein glycosylation requires dolichyl phosphate as a carbohydrate carrier. Dolichols are α-saturated polyprenols, and their saturation in *S. cerevisiae* is catalyzed by polyprenyl reductase Dfg10 together with some other unknown enzymes. The aim of this study was to identify such enzymes in *Candida*. The Dfg10 polyprenyl reductase from *S. cerevisiae* comprises a C-terminal 3-oxo-5-alpha-steroid 4-dehydrogenase domain. Alignment analysis revealed such a domain in two ORFs (orf19.209 and orf19.3293) from *C. albicans*, which were similar, respectively, to Dfg10 polyprenyl reductase and Tsc13 enoyl-transferase from *S. cerevisiae*. Deletion of orf19.209 in *Candida* impaired saturation of polyprenols. The Tsc13 homologue turned out not to be capable of saturating polyprenols, but limiting its expression reduce the cellular level of dolichols and polyprenols. This reduction was not due to a decreased expression of genes encoding *cis*-prenyltransferases from the dolichol branch but to a lower expression of genes encoding enzymes of the early stages of the mevalonate pathway. Despite the resulting lower consumption of acetyl-CoA, the sole precursor of the mevalonate pathway, it was not redirected towards fatty acid synthesis or elongation. Lowering the expression of *TSC13* decreased the expression of the *ACC1* gene encoding acetyl-CoA carboxylase, the key regulatory enzyme of fatty acid synthesis and elongation.

## 1. Introduction

**Figure 1:**
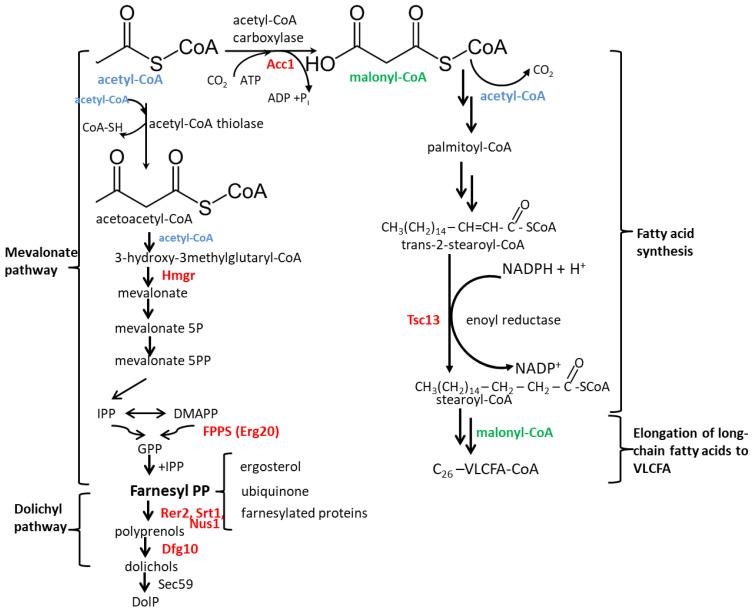
Acetyl-CoA is a common substrate of the mevalonate pathway and the synthesis of very long-chain fatty acids (VLCFA).

Dolichols, α-saturated polyprenols, are synthesized in a branch of the mevalonate pathway called the dolichyl pathway [1]. The starting substrate for the synthesis of dolichols is acetyl–CoA, three molecules of which are converted into a five-carbon intermediate-mevalonate, from which the whole pathway derives its name. In a series of reactions, the mevalonate gives rise to a fifteen-carbon compound, farnesyl diphosphate (FPP) (Figure 1). FPP is a substrate for the synthesis of dolichols, sterols, ubiquinone and other products. To synthesize dolichols, FPP must first be elongated by the addition of isopentenyl diphosphate (IPP) monomers to form a polyprenol. These additions are carried out by *cis*-prenyltransferase (*cis*-PT) (EC 2.5.1.87) [2]. In *Candida albicans*, similar to *S. cerevisiae*, two *cis*-PTs, Rer2 and Srt1 have been found [3,4], each of which form an active complex with Nus1 [5]. In both *S. cerevisiae* and *C. albicans* Rer2 is responsible for about 97% of the activity [3,4]. The α-isoprene unit in the synthesized polyprenol is next saturated to give a dolichol. In *S. cerevisiae*, this reaction is catalyzed by the Dfg10 polyprenyl reductase (EC 1.3.1.94) (saturase) using nicotinamide adenine dinucleotide phosphate (NADPH) as a cofactor [1]. Subsequently, dolichols can be phosphorylated by dolichyl kinase (EC 2.1.108) Sec59 transferring phosphate from cytidine triphosphate (CTP) or can accumulate as free alcohols and/or esters with carboxylic acids [6,7]. The best understood function of dolichols is their participation in protein glycosylation where they serve as carriers/donors of sugar moieties. This function is performed by the phosphorylated form of dolichol, Dol-P.

Hmgr-3-hydroxy-3-methylglutaryl-CoA reductase; IPP-isopentenyl diphosphate; DMAPP-dimethylallyl diphosphate; GPP- geranyl diphosphate; FPPS-farnesyl diphosphate synthase (Erg20); Rer2, Srt1, Nus1-*cis*-prenyltransferases; Dfg10-polyprenyl reductase (saturase); Sec59-dolichyl kinase; Acc1-acetyl-CoA carboxylase; Tsc13-very long chain enoyl-CoA reductase; VLCFA-very long-chain fatty acids.

The mevalonate pathway is not the only biosynthetic route commencing with acetyl-CoA. Another common fate of acetyl-CoA is its carboxylation by biotin-dependent acetyl-CoA carboxylase Acc1 (EC 6.4.1.2) to form malonyl-CoA, a universal precursor of fatty acids, used both for their synthesis de novo and elongation to very long-chain fatty acids (VLCFA) [8,9].

In *S. cerevisiae*, most fatty acids have 16 or 18 carbons. Those up to 16 carbons are synthesized by a soluble cytosolic fatty acid synthase complex (FAS) while the VLCFA (18–26 carbons) are synthesized by an ER membrane-associated fatty acid elongating system; the longest VLCFA species in yeast is hexacosanoic acid, C26 [10,11]. Each VLCFA extension round is catalyzed by an elongation complex combining four activities. The Elo1/2/3 elongases (EC 2.3.1.199) add two-carbon fragments from malonyl-CoA to the fatty acid undergoing elongation and determine the final length of the VLCFA. Ifa38 (EC 1.1.1.330) is the major reductase converting the 3-ketoacyl-CoA formed to 3-hydroxyacyl-CoA which is then processed by the Phs1 3-hydroxyacyl-CoA dehydratase (EC 4.2.1.134) [12]. Finally, Tsc13 enoyl-reductase (EC 1.3.1.93) reduces the double bond to complete the elongation round. The *TSC13* and *PHS1* genes are essential in yeast, while single elongase mutants (Elo1/2/3) show distinct phenotypes accumulating fatty acids of a specific chain length [12]. Since elongation of fatty acids is essential for the cell, a triple Elo mutant is inviable. The deficit of VLCFA cannot be rescued by supplementation, presumably due to insufficient uptake [13].

The VLCFA are key components of sphingolipids, phosphatidylinositols, GPI anchors, and can also be found as a component of storage lipids in lipid droplets (for the role of VLCFA, see review by Erdbrügger and Fröhlich [14]). VLCFA induce membrane curvature because their long chains can span both leaflets of the lipid bilayer, additionally increasing the fluidity of the membranes at high temperatures [14]. 

In this study we show that the biosynthetic pathways leading, respectively, to polyprenols/dolichols and to fatty acids/VLCFA are interdependent in a previously unanticipated manner. When the expression of the *TSC13* gene encoding enoyl-reductase from the latter pathway was decreased we observed not only a downregulation of the *ACC1* gene encoding acetyl-CoA carboxylase from the same pathway, but also a decreased expression of the main regulatory enzymes of the mevalonate pathway, 3-hydroxy-3-methylglutaryl-CoA reductase (Hmgr) and farnesyl diphosphate synthase (Erg20). Those changes were reflected in lower levels of polyprenoids and C18 fatty acids, and an upregulated content of C16 fatty acids.

## 2. Results

### 2.1. C. albicans Orthologues of the S. cerevisiae DFG10 and TSC13 Genes

The aim of this study was to identify *C. albicans* enzymes reducing polyprenols to dolichols. The Dfg10 polyprenyl reductase from *S. cerevisiae* contains a characteristic C-terminal 3-oxo-5-alpha-steroid 4-dehydrogenase domain. This domain was used as a query to identify *C. albicans* genes encoding protein(s) of potential saturase activity.

The *Candida* genome database CGD (www.candidagenome.org accessed on 1 December 2021) contains open reading frames orf19.209 encoding a hypothetical 282 aa protein of 33% identity to the *S. cerevisiae* Dfg10 polyprenyl reductase (saturase) and orf19.3293 encoding a protein of 312 aa showing 25.4% identity to the *S. cerevisiae* steroid 5 α-reductase Tsc13 involved in the elongation of very long chain fatty acids.

**Figure 2:**
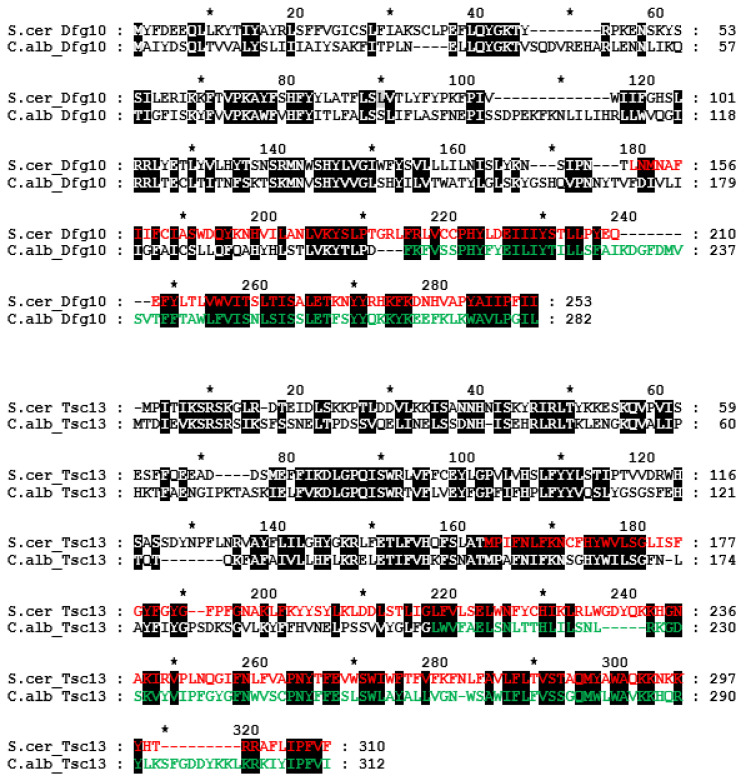
Alignment of putative *C. albicans* Dfg10 and Tsc13 amino acid sequences compared to the *S. cerevisiae* homologues. The 3-oxo-5-alpha-steroid 4-dehydrogenase C-terminal domain is in red and green. Identical amino acids and those of similar chemical character are marked by black background.

Both the putative Dfg10 and Tsc13 homologues not only contain a C-terminal 3-oxo-5-alpha-steroid 4-dehydrogenase domain (Pfam) [15], but additionally show marked homology along their entire length, which suggests they could have similar functions (Figure 2).

**Figure 3:**
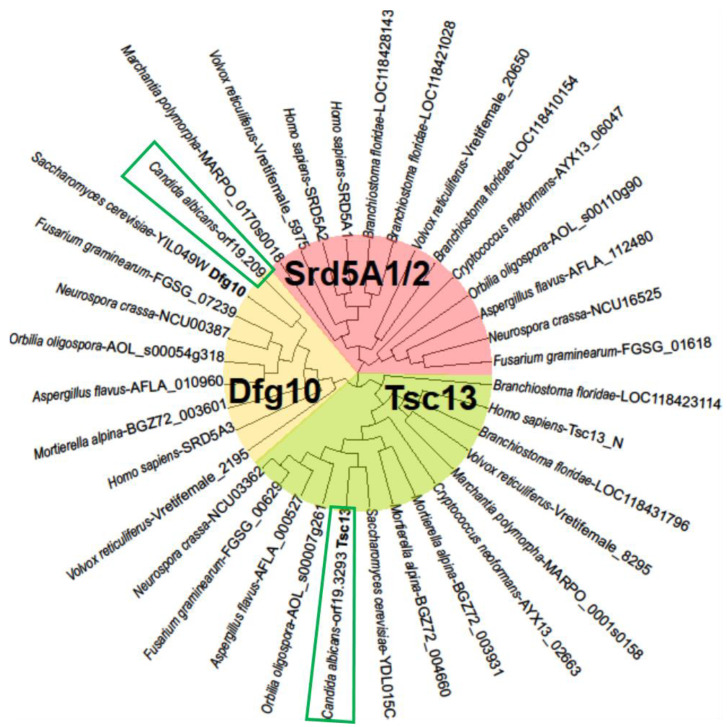
Phylogenetic tree of homologues of Dfg10 and Tsc13 proteins from *S. cerevisiae* and SRD5A1 from human. The proteins carry the PF 02544 Pfam domain of 3-oxo-5-alpha-steroid 4-dehydrogenase.

On the other hand, human SRD5A1 and SRD5A2 steroid 5α reductases [16] also comprising C-terminal 3-oxo-5-alpha-steroid 4-dehydrogenase domain have no homologues in *C. albicans* and *S. cerevisiae* while they were revealed for filamentous fungi (Figure 3, pink background). Phylogenetic analysis showed that the *C. albicans* proteins encoded by orf.19.209 and orf.19.3293 are closely related to Dfg10 and Tsc13 from *S. cerevisiae*, respectively (Figure 3, yellow and green background).

To examine the functions of the hypothetical Dfg10 and Tsc13 proteins, we deleted both copies of the *DFG10* gene in the diploid CAI4 strain of *C. albicans*, or one copy of *TSC13* with the second one placed under the *MET3* regulatory promoter (deletion of both *TSC13* alleles turned out to be lethal, data not shown). The latter construct allowed manipulation of the *TSC13* expression level by growing the strain under non-repressing conditions (normal medium) or under repression (methionine and cysteine added to the medium see Material and Methods).

Construction of the mutants was verified by Southern blotting (Supplementary Figure S1).

**Figure 4:**
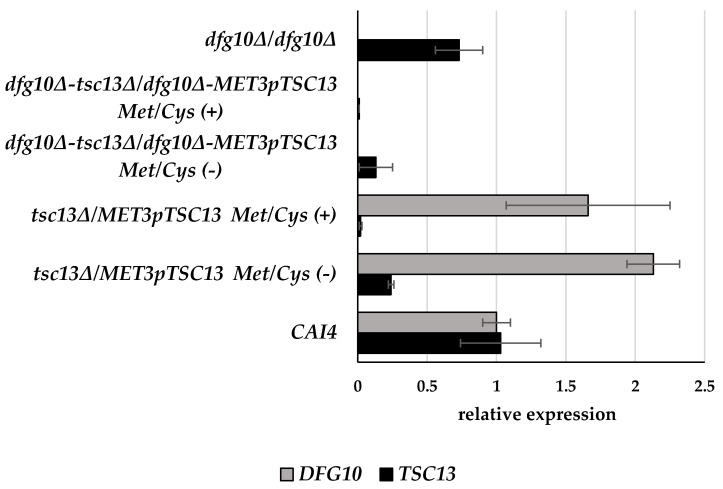
Transcript levels of *DFG10* and *TSC13* genes in wild-type and mutant *C. albicans* strains. *C albicans* was grown in normal medium (Met/Cys (−)) or under conditions repressing the *MET3* promoter (Met/Cys (+)). mRNA levels of genes of interest were determined by RT-qPCR relative to *ACT1* mRNA. Data are mean ± SD from three independent experiments, each determined in triplicate.

To quantify expression of *DFG10* (orf19.209) and *TSC13* (orf19.3293) in the wild-type and mutant strains, total RNA was isolated and subjected to RT-qPCR analysis. As expected, no *DFG10* transcript was detected in the *dfg10∆/dfg10∆* strain. In the *tsc13∆/MET3*p*TSC13* strain grown under non-repressing conditions (without Met or Cys in the medium) the level of *TSC13* mRNA was ca. 24% of that in the wild type, while upon repression, the transcript could no longer be detected (Figure 4). Nevertheless, the level of *TSC13* expression was apparently sufficient to support growth under these conditions.

Unexpectedly, in the *tsc13∆/MET3*p*TSC13* mutant, expression of the *DFG10* gene was elevated significantly (by ca. 66–113%) compared to the wild-type strain, in both the repression and derepression conditions.

Polyprenoid abundance in C. albicans strains with Dfg10 and/or Tsc13 deficits.

In *S. cerevisiae* the Dfg10 protein is engaged in the saturation of the α isoprene unit of polyprenols converting them to dolichols. Bearing in mind the substantial homology of the putative *C. albicans* Dfg10 to its *S. cerevisiae* counterpart, we expected a shift in the ratio between dolichols and polyprenols towards the latter in the *dfg10∆/dfg10∆* mutants of *C. albicans*.

**Figure 5:**
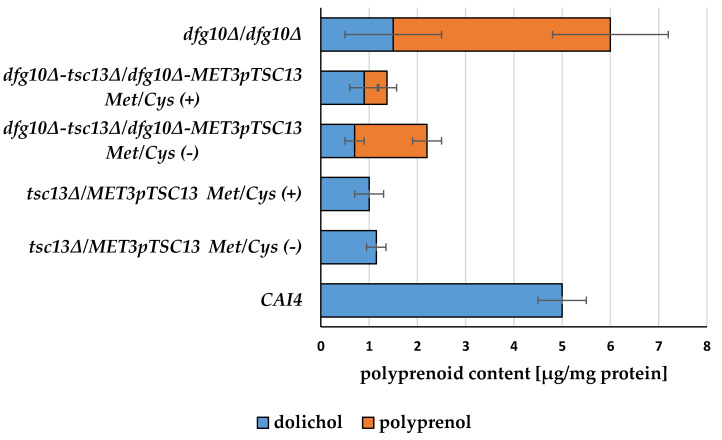
Polyprenoid content in wild-type *C.* *albicans* and *dfg10* and *tsc13* mutants.

To verify this assumption, we analyzed the content of polyprenols and dolichols in the respective mutants relative to those in the wild type (Figure 5).

*C. albicans* was grown in normal medium (Met/Cys (−)) or under conditions repressing the *MET3* promoter (Met/Cys (+)).

Data are mean ± SD from three independent experiments, each determined in triplicate.

As expected, the wild-type strain CAI4 contained exclusively dolichols, while in the *dfg10∆/dfg10∆* strain as much as ca. 75% of the polyprenoids were not reduced, i.e., were polyprenols and only 25%-dolichols. This dramatic shift confirms that indeed the *C. albicans* gene (orf.19.209), tentatively designed as *DFG10,* encodes a polyprenol α saturase. Interestingly, the overall content of polyprenoids (i.e., polyprenols and dolichols combined) was ca. 20% higher in the *dfg10∆/dfg10∆* strain than that in the wild type.

Since some dolichols were still present in the *dfg10∆/dfg10∆* strain, one must conclude that Dfg10 is not the only polyprenol reductase.

In principle, that activity could be provided by steroid 5α-reductase Tsc13, although it has been reported that Tsc13 is unlikely to catalyze this reaction in *S. cerevisiae* since no change in the dolichol/polyprenol ratio was observed upon *TSC13* deletion [17].

In contrast, our analysis of polyprenoids in *C. albicans* mutants (Figure 5) showed a rather complex effect of lowering expression of the *TSC13* gene on the cellular content of both polyprenols and dolichols. In the *tsc13∆/MET3*p*TSC13* strain the cellular content of dolichols was only ca. 20% of that in the wild type, and polyprenols were still absent. Interestingly, the amount of dolichols was nearly identical, regardless of the expression level of the *MET3*p driven copy of *TSC13*, that is, relatively high (ca. 24% of wild-type value) in non-repressive conditions or virtually undetectable under Met/Cys repression.

A lowered expression of *TSC13* in the *dfg10∆/dfg10∆-tsc13∆/MET3*p*TSC13* mutant decreased the content of dolichols even further, by ca. 30–50% relative to that in *dfg10∆/dfg10∆*, but also profoundly decreased the abundance of unreduced polyprenols, to ca. 30% of that found in *dfg10∆/dfg10∆* without repression of the *MET3* promoter and ca. 12% when the expression of *MET3*p*TSC13* was repressed. As a consequence, the overall content of polyprenoids decreased to 37% and 23% of that in *dfg10∆/dfg10∆-tsc13∆/MET3*p*TSC13* under, respectively, non-repressing and repressing conditions.

Taken together, these results show that while also in *C. albicans*, Tsc13 does not seem to catalyze α saturation of polyprenols, its high expression is required to allow their efficient synthesis.

### 2.2. Gene Expression in DFG10 and TSC13 Mutants

Since we observed a decreased level of dolichols in strains with lowered expression of the *TSC13* gene, we asked whether that effect could be due to a changed expression of genes coding for the *cis*-prenyltransferases Rer2, Srt1 and Nus1 engaged in dolichol biosynthesis.

**Figure 6:**
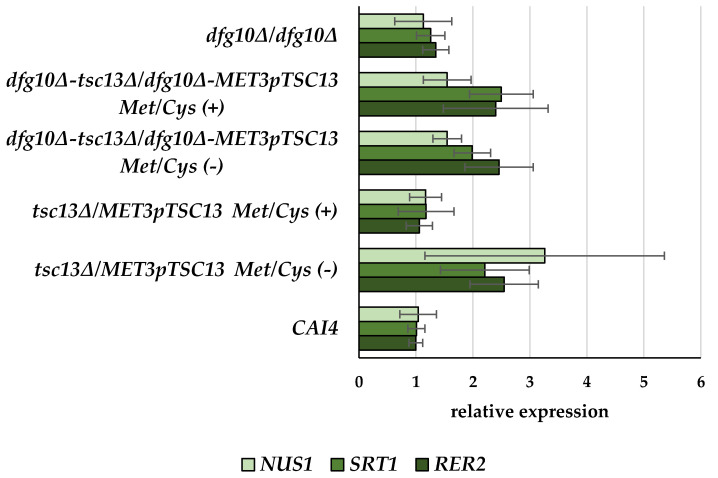
Transcript levels of *NUS1*, *SRT1* and *RER2* genes in wild-type and mutant *C. albicans* strains. *C albicans* was grown in normal medium (Met/Cys (−)) or under conditions repressing the *MET3* promoter (Met/Cys (+)). mRNA levels of genes of interest were determined by RT-qPCR relative to *ACT1* mRNA. Data are mean ± SD from three independent experiments, each determined in triplicate.

Expression of the *RER2*, *SRT1* and *NUS1* genes was not affected when the expression of *TSC13* was almost fully prevented (*tsc13∆/MET3*p*TSC13* in repressing conditions) nor in the *dfg10∆/dfg10∆* strain (Figure 6). However, a moderate decrease in *TSC13* expression alone, and also a combination of the *dfg10∆/dfg10∆* mutation with a decreased (moderately or strongly alike) expression of *TSC13* all resulted in a ca. 2-fold elevation of expression of all three *cis*-prenyltransferase-encoding genes. Thus, there seems to be no straightforward correlation between the level of dolichols (or polyprenoids in general) in the *dfg10* and *tsc13* mutants and the expression of the enzymes carrying out the elongation of the polyprenyl chain.

**Figure 7:**
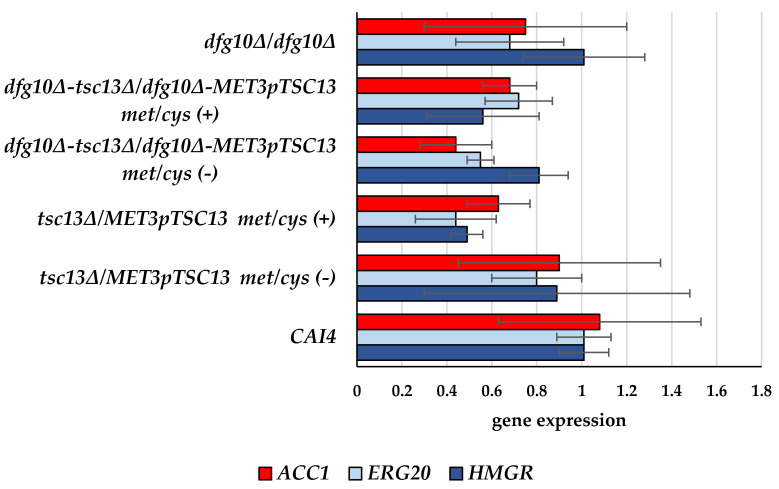
Transcript levels of ACC1, ERG20 and HMGR genes in wild-type and mutant *C. albicans* strains. C albicans was grown in normal medium (Met/Cys (−)) or under conditions repressing the MET3 promoter (Met/Cys (+)). mRNA levels of genes of interest were determined by RT-qPCR relative to ACT1 mRNA. Additionally, we considered a wholly different mode of down-regulation of dolichol biosynthesis—by depletion of its precursor, acetyl-CoA. Following this reasoning, we determined the expression of the *ACC1* gene encoding acetyl-CoA carboxylase, the first and limiting enzyme of the fatty acid synthesis/elongation pathways [8]; its increased activity would enhance consumption of acetyl-CoA, thereby limiting its availability for the mevalonate pathway and further for the synthesis of polyprenoids. Data are mean ± SD from three independent experiments, each determined in triplicate.

Since no regulation specific to the dolichol branch could be shown, we reasoned that an earlier step(s) could be affected, i.e., the mevalonate pathway itself. To verify this possibility, we determined the expression of two known regulatory genes of the mevalonate pathway, *HMGR* (encoding 3-hydroxyl-3-methylglutaryl-CoA reductase) and *ERG20* (encoding farnesyl diphosphate synthase) (Figure 7).

We found a moderate down-regulation of expression of both regulatory genes of the mevalonate pathway, reaching ca. 50% in the *tsc13∆/MET3*p*TSC13* strain grown under repressing conditions, which could explain, at least in part, its lower content of polyprenoids.

The *ACC1* gene was also moderately down-regulated, refuting our hypothesis regarding competition for acetyl-CoA between those two biosynthetic pathways.

### 2.3. Fatty Acid Content in Membranes of DFG10 and TSC13 Mutants

Malonyl-CoA is also used for the synthesis of very long chain fatty acids catalyzed by the ER membrane-bound fatty acid elongation system containing Tsc13. This enzymatic complex elongates palmitic acid (C16) to stearic acid (C18) and then to longer species.

**Figure 8:**
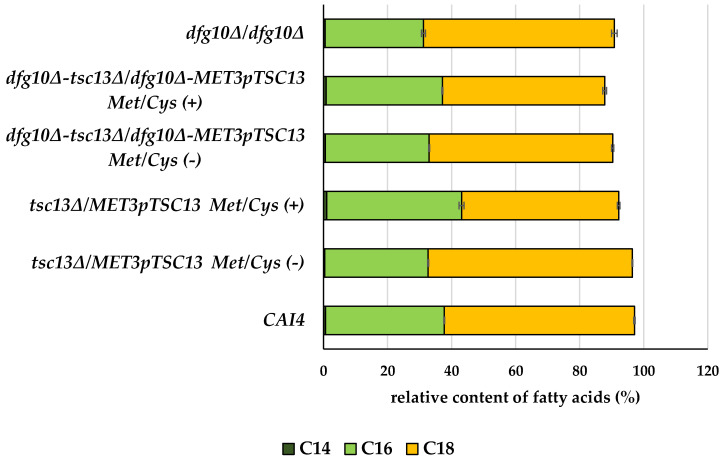
Relative abundance of C18, C16 and C14 fatty acids in membrane fraction of wild-type and mutant *C. albicans* strains. *C albicans* was grown in normal medium (Met/Cys (−)) or under conditions repressing the *MET3* promoter (Met/Cys (+)). Analysis of fatty acids was conducted with an Agilent Model 7890 gas chromatograph equipped with a 5975C mass detector. Data are mean from three independent experiments, each determined in triplicate.

Having observed a decreased expression of *ACC1* in the *dfg10/tsc13* mutants we asked whether it could affect fatty acid elongation. To this end we analyzed the profile of membrane fatty acids in all the mutants (Figure 8; Table S1).

C14 corresponds to the saturated meristic acid only (C14:0), C16 comprises palmitic (C16:0) and palmitoleic (C16:1) acids, and C18-stearic (C18:0), oleic (C18:1), linoleic (C18:2) and linolenic (C18:3) acids.

The values may not add up to 100%, as other fatty acid species were not included in the calculation. For details see Table S1.

The level of C18 fatty acids was 17.4% lower in the *tsc13∆/MET3*p*TSC13* mutant grown in the repression conditions compared to the wild-type strain. *DFG10* depletion did not influence the abundance of C18 fatty acids when compared to the control strain.

### 2.4. Defective Saturation of Polyprenols and Fatty Acid Elongation Alter Morphology of C. albicans

*C. albicans* can grow in a unicellular form but also as pseudohyphae and true hyphae. A genetic screen of *S. cerevisiae* for mutant strains defective in filamentous growth (dfg), has identified several mutations hampering filamentous growth, including *dfg10* [18]. It has been shown that *C. albicans* strains defective in dolichol synthesis cannot grow in the filamentous (hyphal) form [4]. Since our mutants were disturbed in dolichol synthesis, we tested their hyphae-forming ability by cultivating them on horse serum medium (YPSerum) and on Spider medium.

**Figure 9:**
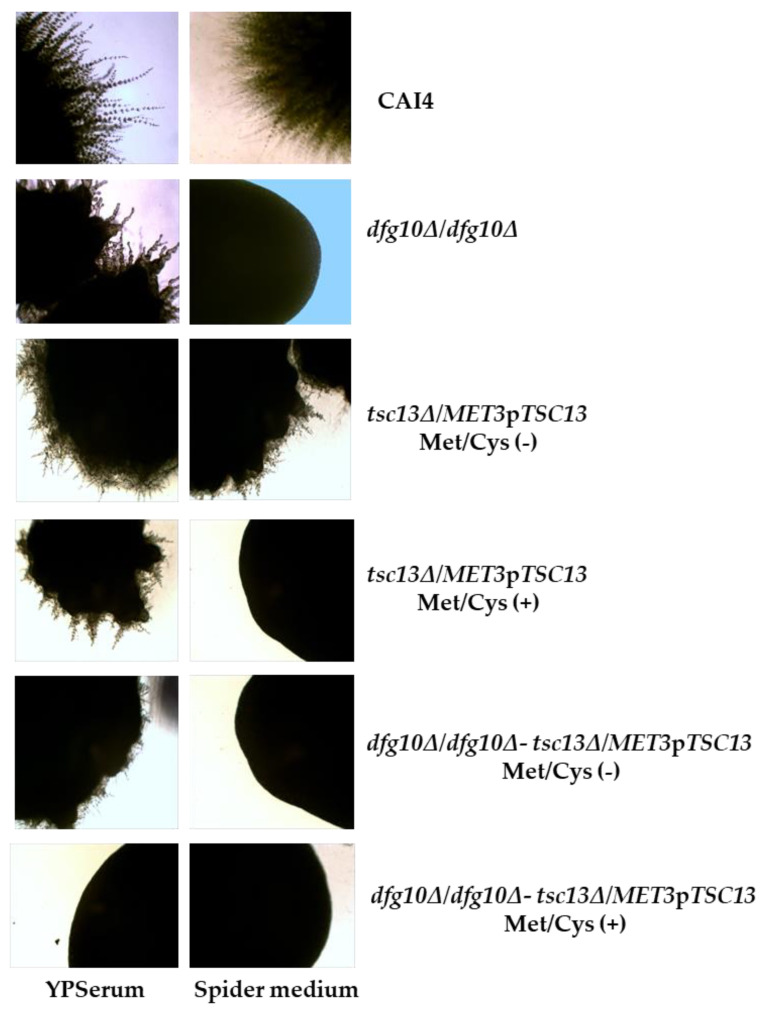
Hyphal growth of wild-type and mutant *C. albicans.* Hyphae formation was induced for 7 days at 30 °C on YPSerum and Spider medium plates supplemented or not with methionine and cysteine, as indicated. Colonies were photographed under a light microscope at 10× magnification.

The wild-type strain efficiently formed filaments on both Spider medium and YPSerum while the *dfg10∆/dfg10∆-tsc13∆/MET3*p*TSC13* mutant was unable to do so on either medium supplemented with methionine and cysteine (Figure 9). The ability to form hyphae by the other mutants varied depending on the medium used and/or the extent of *TSC13* expression (i.e., the presence of Met/Cys in the medium (Figure 8)).

## 3. Discussion

In this study, we characterized two previously uncharacterized genes of *C. albicans*, orf19.209 and orf19.3293. They were found to encode proteins showing moderate homology to the Dfg10 and Tsc13 proteins from *S. cerevisiae*.

The *C. albicans* Dfg10 showed the same activity as its *S. cerevisiae* counterpart, i.e., reduction of polyprenols to dolichols. However, in both the *∆dfg10/∆dfg10* mutant of *Candida* (this study), as well as in the *S. cerevisiae dfg10–100* mutant [17], dolichols were still present at ca. 30% of their normal content, indicating that in both yeast species Dfg10 is not the only enzyme capable of α-saturating polyprenols. 

Cantagrel et al. [17] suggested that the Tsc13 steroid 5α-reductase could be a good candidate for an additional enzyme saturating polyprenols in *S. cerevisiae*; however, their further experiments ruled out that possibility as they found that the polyprenol/dolichol ratio was unchanged in the double *dfg10/tsc13* mutant of *S. cerevisiae*. Unexpectedly, in light of those results, a lowered expression of the *TSC13* gene homologue (*tsc13∆/MET3*p*TSC13* Met/Cys (+)) in *C. albicans* caused a profound decrease in the dolichol level, even though the expression of the *RER2* and *SRT1* genes encoding *cis*-prenyltransferases was not changed. An increased *TSC 13* expression in the mutant cultured without methionine/cysteine, but still well below that in the wild type, markedly increased *RER2* and *SRT1* expression, but the dolichol level remained low. Independently of the cultivation conditions, expression of the *DFG10* gene was increased in this mutant compared with the wild type. Since dolichols, and not polyprenols, have the ability to carry carbohydrates during protein glycosylation, saturation of the polyprenols is essential [19]. Therefore, the significantly depleted pool of polyprenol in the *tsc13∆/MET3*p*TSC13* mutant should be fully saturated to dolichols. Simultaneously, the decreased *TSC13* expression in the *tsc13∆/MET3*p*TSC13* strain (Met/Cys (+)) decreased the expression of the regulatory genes of the mevalonate pathway (*HMGR* and *ERG20*).

Inhibition of the mevalonate pathway could be expected to direct the unconsumed acetyl-CoA to the synthesis of malonyl-CoA by Acc1 and thereby support elongation of fatty acids, but our *tsc13* mutant (with or without an accompanying deletion of *DFG10*) revealed lower expression of the *ACC1* gene (Figure 7). Tsc13 catalyzes the elongation of palmitoyl-CoA utilizing malonyl-CoA and accordingly in the *tsc13∆/MET3*p*TSC13* mutant (Met/Cys (+)), the ratio between C16 and C18 fatty acids was increased, albeit the depletion of the latter was only moderate. A preferential use of malonyl-CoA for the elongation of fatty acids has been reported [11,20]. It was shown that a residual malonyl-CoA production in an *acc1* mutant appears sufficient to sustain VLCFA synthesis, suggesting a preferential channeling of malonyl-CoA to the elongation complex in the endoplasmic reticulum. This, in fact, underlines the vital role of very long-chain fatty acids as essential components of sphingolipids, GPI-anchors, phospholipids and storage lipids [14].

On the other hand, the elongation requires both malonyl-CoA and the C16 fatty acid as substrates, so the synthesis of fatty acids by the cytosolic synthase complex must be well balanced with their elongation to VLCFA in the ER.

The synthesis of polyprenols and the elongation of fatty acids must be well regulated in the *dfg10∆/dfg10∆-tsc13∆/MET3*p*TSC13* mutant, as both end-products of the manipulated pathways are essential. An inhibition of expression of *TSC13* combined with no *DFG10* expression elevated the expression of *RER2* and *SRT1* relative to the wild-type level and slightly increased the amount of polyprenoids in comparison with the single *tsc13∆/MET3*p*TSC13* mutant.

A regulation of fatty acid synthesis by products of the mevalonate pathway has already been reported [21]. Geranylgeranyl diphosphate (GGPP) inhibited while oxysterol stimulated fatty acid synthesis; the addition of mevalonate or farnesyl diphosphate (FPP) prevented this activation. FPP was also found to regulate fatty acid synthesis independently of the oxysterol regulatory mechanism [22]. Whether the isoprenoids affected the expression of genes involved in fatty acid biosynthesis or acted via different mechanisms was not determined.

Our present results showed that very low expression of *TSC13* inhibits expression of the regulatory genes of the mevalonate pathway, *HMGR* and *ERG20*, simultaneously with the expression of *ACC1*. Since both dolichols and VLCFA are essential for the cell, a concerted down-regulation of both biosynthetic routes allows the balance between them to be preserved.

## 4. Materials and Methods

### 4.1. Strains and Growth Conditions

*C. albicans* strain CAI4 (genotype: *ura3∆::imm434/ura3∆::imm434*), a uridine auxotroph, was used for deletion of orf19.209 (*DFG10*) and orf19.3293 (*TSC13*).

*C. albicans* strains were routinely grown on YPD medium (1% yeast extract, 1% bacto-peptone, 2% glucose) or SD medium (0.67% yeast nitrogen base, 2% glucose) supplemented with uridine when required. The *MET3* promoter was induced on media supplemented with 2.5 mM methionine and cysteine (Met/Cys) [23].

Spider medium (1% nutrient broth, 1% mannitol, 0.2% K_2_HPO_4_, 1.35% agar) and YPSerum (1% yeast extract, 0.5% bacto-peptone, 10% horse serum, 2% agar) were used for testing hyphae formation [24]. FOA plates (0.67% yeast nitrogen base, 1% casamino acids, 2% glucose, 0.3% 5-fluoroorotic acid, 40 μg/mL uridine, 2% agar) were used to force the excision of the *URA3* gene from *C. albicans* transformants.

*E. coli* strain DH5αF’ (*F’ supE44 ΔlacU169 {ϕ80 lacZΔM15} hsdR17 recA1 endA1 gyrA96 thi-1 relA1*) was used for plasmid propagation and DNA cloning. *E. coli* was grown at 37 °C in liquid or solid LB medium (1% bacto-peptone, 0.5% yeast extract, 1% NaCl, solidified with 2% agar) supplemented with ampicillin (100 µg/mL) when indicated.

### 4.2. Construction of Candida albicans Strains

The *dfg10∆/dfg10∆* mutant was constructed by the URA-blaster method [25]. To construct the *DFG10* deletion cassette, we used the following pairs of primers (Table S2): DFG10F1F/ DFG10F1R to amplify 5′ region of homology (−315 to −16, with reference to the first nucleotide in ORF) and DFG10F2F/DFG10F2R to amplify 3′ region of homology (−306 to −7, with reference to the last nucleotide in ORF). The fragments were cloned into the SacI/BglII and BamHI/PstI restriction sites in p5921 [25], respectively. The URA-blaster cassette flanked by 299-bp upstream and 299-bp downstream sequences complementary to *DFG10* was released by digestion with SacI and PstI and used for sequential replacement of the two *DFG10* alleles in CAI4. The *URA3* selective marker was removed by selection on FOA plates.

The cassette for *TSC13* deletion was constructed with primer pairs TSC13F1F/TSC13F1R and TSC13F2F/TSC13F2R to amplify the 5′ (−213 to +85, with reference to the first nucleotide in ORF) and 3′ regions of homology (−301 to −1, with reference to the last nucleotide in ORF). The amplified fragments were cloned into the SacI/BglII and BamHI/PstI restriction sites in p5921, respectively. The URA-blaster cassette flanked by 298-bp upstream and 300-bp downstream sequences complementary to *TSC13* was cleaved by digestion with SacI and PstI and transformed into CAI4 and *dfg10Δ/dfg10Δ* strains to generate respective heterozygous strains. The *URA3* selective marker was removed by selection on FOA plates.

The correct cassette integration was verified by PCR and Southern blotting.

To place the second allele of *TSC13* under the control of the *MET3* promoter, primers TSC13MET-F and TSC13MET-R were used to amplify the promoter cassette on the template of plasmid pCaDis [23]. The fragment contained: a 65-bp fragment of homology to sequence −317 to −252 bp upstream of *TSC13* start codon, the *URA3* selection marker, the regulatable *MET3* promoter, and a 63-bp fragment of homology to sequence from the first nucleotide of ATG codon to +63 bp downstream the start codon of *TSC13*. The cassette was used for transformation of the *tsc13∆/TSC13* and *dfg10∆/dfg10∆- tsc13∆/TSC13* strains. The *C. albicans* strains constructed in the present study are described in Table 1. 

### 4.3. RNA Isolation, RT-qPCR

Total RNA was isolated using the single-step method described by Chomczynski and Sacchi [26] and cleaned from residual DNA with Clean-Up RNA Concentrator (A&A Biotechnology). RNA concentration was determined with a DS-11 UV-Vis spectrophotometer (DeNovix). The RNA was reverse transcribed in a Civic Cycler Ver II Thermocycler (Biogenet) using the PrimeScript 1st strand cDNA Synthesis Kit (Takara). For qPCR, 20 ng of cDNA was mixed with 0.2 micromoles of forward and reverse primers and 7.5 μL of Sybr Green JumpStart Taq Ready Mix (Merck) in a 96-well plate and the reaction was run in a LightCycler^®^ 96 (Roche Diagnostics GmbH) using the following steps: initial denaturation at 94 °C for 120 s, 40 cycles of denaturation at 94 °C for 15 s, and annealing/extension at 60 °C for 60 s. Three technical repeats were carried out for each data point. The Cq values were calculated automatically by the instrument software. The delta–delta Ct method (∆∆Ct) was used to calculate the relative level of a gene transcript [27]. The NormFinder web tool was used for reference gene validation [28] according to the method described previously [29]. Among the genes tested, *ACT1* appeared to be the most stable and was used as the reference gene. The primers used presented in Table S3 were designed using the Primer Blast web tool [30] and tested in silico using Sequence Manipulation Suite: PCR Primer Stats [31]. Primer efficiency (%) was calculated from standard curves.

### 4.4. Extraction and Purification of Polyisoprenoids

*C. albicans* biomass was harvested by filtration, suspended in 60% KOH with 0.25% pyrogallol and hydrolyzed at 100 °C for 1 h. Lipophylic products were extracted with diethyl ether and evaporated to dryness, then dissolved in hexane and applied onto a silica gel column equilibrated with hexane. The column was washed with 3% diethyl ether in hexane and the polyisoprenoid fraction was eluted with 10% and 20% diethyl ether in hexane, the two eluates were pooled, evaporated to dryness, dissolved in isopropanol and subjected to HPLC/UV analysis as described earlier [32], with modifications. Briefly, a linear gradient of methanol: water (9:1, *v*/*v*) in methanol:isopropanol:hexane (2:1:1, *v*/*v*/*v*) was used for elution. Fractions containing polyisoprenoids (polyprenols and dolichols) were collected (based on comparison of their retention times with those of yeast dolichols used as external standards) and their structure was analyzed (see below). For quantitative analysis, Dol13 was added to the biomass as an internal standard before hydrolysis. The polyprenol and dolichol standards were from the Collection of Polyprenols (IBB PAS).

### 4.5. Lipid Analysis

*C. albicans* biomass was harvested and lyophilized. Then, 100 mg of biomass powder was put into 2-mL tubes (Eppendorf) containing glass beads, 0.66 mL of chloroform and 0.33 mL of methanol [33]. The samples were homogenized using a FastPrep-24 homogenizer at a vibration frequency of 5 m s^−1^ for 120 s. The mixture was transferred to another tube and 0.2 mL of 0.9% saline was added. Fractions were separated by 30 s centrifugation at 2000× *g*. The lower layer was collected without the beads and cell debris and evaporated, dissolved in 1.5 mL of methanol and transferred to a screw-capped glass test tube. To this solution, 0.2 mL toluene and 0.3 mL of 8% HCl in methanol were added [34]. The tube was vortexed and incubated overnight at 45 °C. After cooling to room temperature, 1 mL of hexane and 1 mL of deionized water were added for the extraction of fatty acid methyl esters (FAMEs). The tube was vortexed, and 0.2 mL of the hexane layer was removed. The FAMEs were separated using an Agilent Model 7890 gas chromatograph equipped with a 5975C mass detector and an HP 5 MS methyl polysiloxane capillary column (30 m × 0.25 mm i.d. × 0.25 mm ft) with helium as a carrier gas. The temperature of the column was maintained at 60 °C for 3 min, then increased to 212 °C at the rate of 6 °C min^−1^, followed by an increase to 245 °C at the rate of 2 °C min^−1^, and, finally, to 280 °C at the rate of 20 °C min^−1^, at which point it was held for 10 min. Split injection at 250 °C was employed. The FAMEs were identified by comparison with authenticated reference standards (Sigma, Supelco).

### 4.6. Phylogenetic Analysi

Selection of protein dataset: sequences of well-characterized yeast Dfg10 (Acc. no. NP_012215, locus YIL049W), Tsc13 (Acc. no. NP_010269, locus YDL015C) and human SRD5A1 (Acc. no. NP_001038, locus NC_000005.10) were queried against the GenBank nonredundant protein database using BLAST [35]. All proteins with the PF 02544 Pfam domain of 3-oxo-5-alpha-steroid 4-dehydrogenase were extracted and subjected to further analysis. Each taxonomic group among animals, plants and fungi was represented by at least two species. Sequences were aligned with MAFFT v7.409 using the automate strategy [36] and trimmed manually. The phylogenetic neighbor-joining tree was computed with PhyML v3.0 using the automatic model selection criterion SMS [36] and the Akaike information criterion. The cladogram was visualized by PRESTO (a Phylogenetic tReE viSualizaTiOn) (http://www.atgc-montpellier.fr/presto accessed on 1 December 2021) from The ATGC Montpellier Bioinformatics Platform.

## 5. Conclusions

This study showed that decreased expression of *TSC13* gene encoding enoyl-reductase from the biosynthetic pathway of fatty acids/VLCFA reduced the level of polyprenoids synthesized in the dolichyl branch of the mevalonate pathway.

This reduction was due to a lower expression of genes encoding enzymes of the early stages of the mevalonate pathway. Simultaneously, expression of the *ACC1* gene encoding acetyl-CoA carboxylase, the key regulatory enzyme of fatty acid synthesis and elongation, was also reduced.

We conclude that down-regulation of both biosynthetic pathways keeps them in a proper balance.

## Figures and Tables

**Table 1:** *C. albicans* strains used in this study.

Strain	Genotype	Source
CAI-4	*ura3Δ::im434/ura3Δ::imm434*	[26]
*dfg10(URA3)/DFG10*	CAI4; *dfg10Δ::hisG-URA3-hisG/DFG10*	This study
*dfg10∆/DFG10*	CAI4; *dfg10Δ::hisG/DFG10*	This study
*dfg10(URA3)/dfg10∆*	CAI4 but *dfg10Δ::hisG/dfg10Δ:hisG-URA3-hisG*	This study
*dfg10∆/dfg10∆*	CAI4; *dfg10Δ:hisG/dfg10Δ::hisG*	This study
*tsc13(URA3*)/*TSC13*	CAI4; *tsc13Δ::hisG-URA3-hisG/TSC13*	This study
*tsc13∆/MET3pTSC13*	CAI4; *tsc13Δ::hisG/MET3*p*TSC13*	This study
*dfg10∆-tsc13∆/dfg10∆-MET3pTSC13*	CAI4; *dfg10Δ:hisG/dfg10Δ::hisG- tsc13Δ::hisG/MET3*p*TSC13*	This study

## Data Availability

Not applicable.

## Supplementary Materials

The following supporting information can be downloaded at: www.mdpi.com/article/10.3390/ijms23010409/s1.
