# Supplementary material for: Synthesis of Dolichols in Candida albicans Is Co-Regulated with Elongation of Fatty Acids
Source: Int J Mol Sci. 2021 Dec 30;23(1):409. doi: 10.3390/ijms23010409 (PMC8745096; doi:10.3390/ijms23010409)
Supplement: Supplementary file 1 [file ijms-23-00409-s001.zip › ijms-1526644-supplementary.pdf]

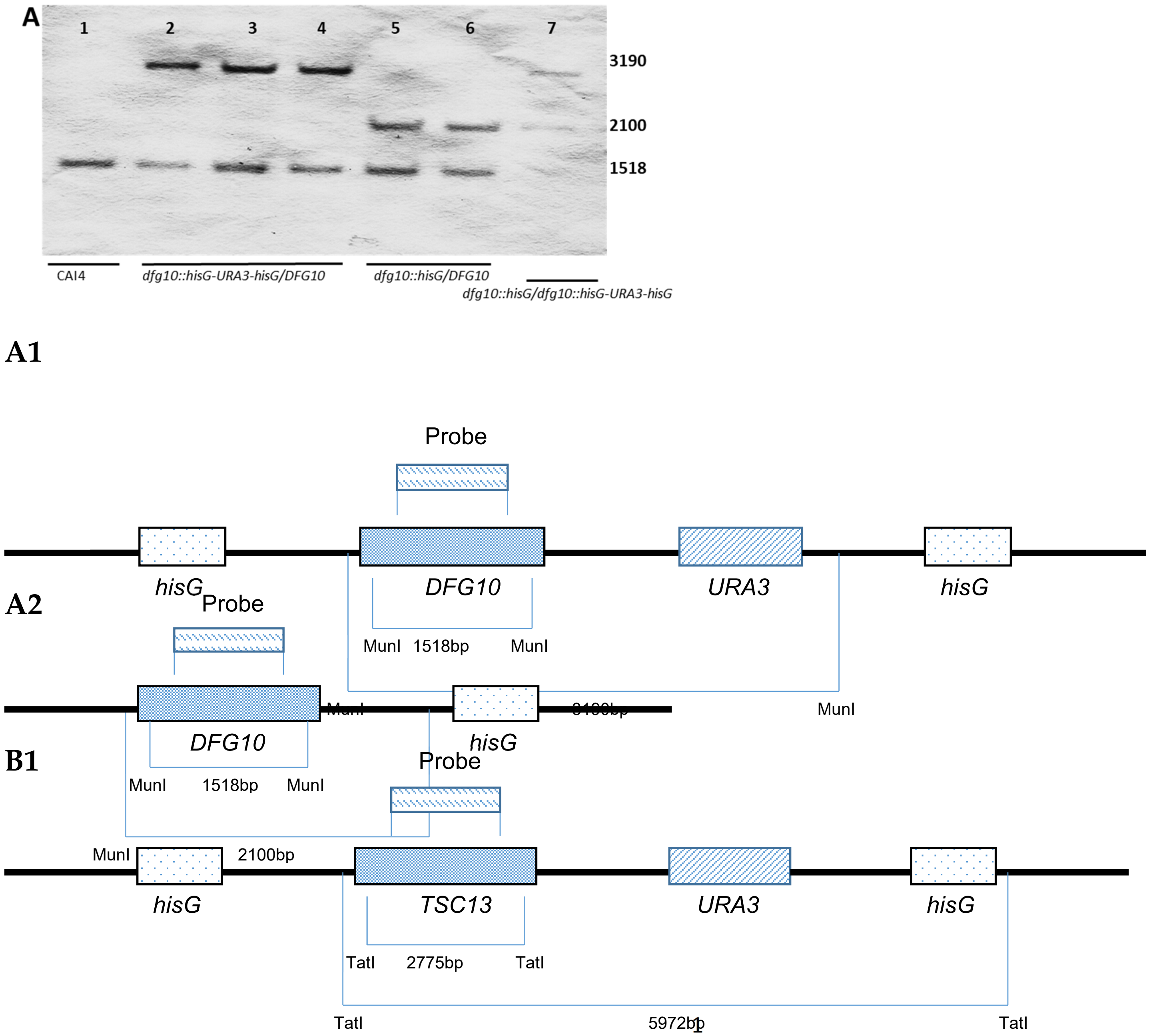
A
1
2
3
4
5
6
7
3190
2100
1518
CAI4
dfg10::hisG-URA3-hisG/DFG10
dfg10::hisG/DFG10
dfg10::hisG/dfg10::hisG-URA3-hisG
A1
Probe
hisG
DFG10
URA3
hisG
MunI
1518bp
MunI
MunI
A2
Probe
DFG10
MunI
hisG
2100bp
MunI
1518bp
MunI
B1
Probe
MunI
hisG
2100bp
TSC13
URA3
hisG
TatI
2775bp
TatI
TatI
5972bp
TatI

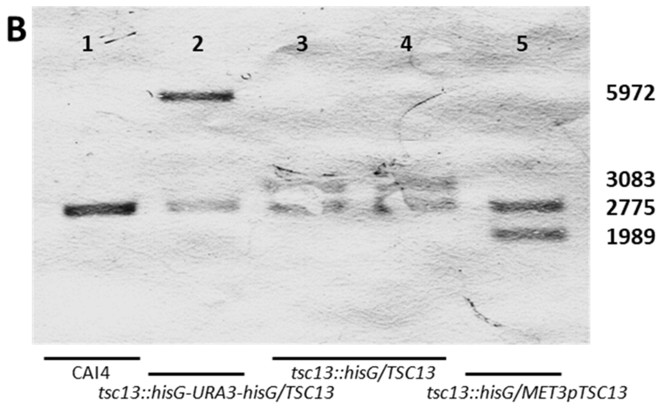
B
1
2
3
4
5
5972
3083
2775
1989
CAI4
tsc13::hisG-URA3-hisG/TSC13
tsc13::hisG/TSC13
tsc13::hisG/MET3pTSC13

B2

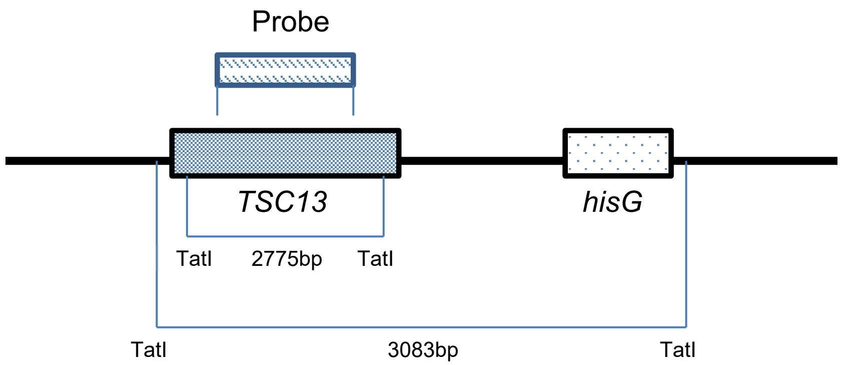
Probe
TSC13
hisG
TatI
2775bp
TatI
TatI
3083bp
TatI

B3

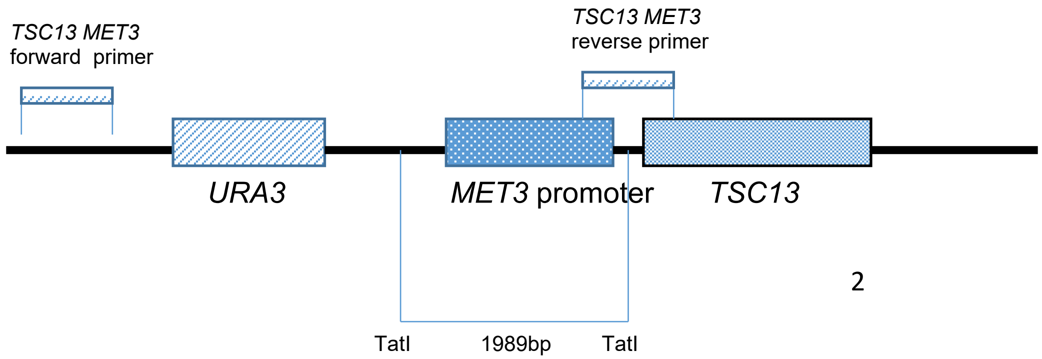
TSC13 MET3
forward primer
TSC13 MET3
reverse primer
URA3
MET3 promoter
TSC13
TatI
1989bp
TatI

Figure S1. Southern analysis of *dfg1Δ0* (A) and *tscΔ13* (B) strains of *C. albicans* CAI4.

A- Analysis of *dfg10Δ* strain
MunI-digested DNA of control strain CAI4 (lane1), hemizygote *dfg10::hisG-URA3-hisG/DFG10* before *URA3* excision (lane 2, 3, 4) hemizygote *dfg10::hisG/DFG10* after *URA3* excision (lane 5, 6), homozygote *dfg10::hisG/dfg10:hisG-URE3-hisG* strain (lane 7)
Blot was probed with digoxigenin-labeled *DFG10* fragment and visualized with NBT/BCIP

A1- restriction map of *DFG10* region before *URA3* excision
A2- restriction map of *DFG10* region after *URA3* excision

B- Analysis of *tsc13Δ*
TatI-digested DNA of control strain CAI4 (lane1), hemizygote *tsc13::hisG-URA3-hisG/TSC13* before *URA3* excision (lane 2), hemizygote *tsc13::hisG/TSC13* after *URA3* excision, *tsc13::hisG/MET3*p*TSC13* strain (lane 5).
Blot was probed with digoxigenin-labeled *TSC13* fragment and visualized with NBT/BCIP

B1- restriction map of *TSC13* region before *URA3* excision
B2- restriction map of *TCS13* after *URA3* excision
B3- restriction map of *TSC13* under the regulatable *MET3* promoter

Table S1

Profile of fatty acids in membranes from wild type and mutants of *C. albicans* strains

	CAI4	*tsc13Δ/MET3pTSC13* (Met/Cys -)	*tsc13Δ/MET3pTSC13* (Met/Cys +)	*dfg10Δ/dfg10Δ-tsc13Δ/MET3pTSC13* (Met/Cys -)	*dfg10Δ/dfg10Δ-tsc13Δ/MET3pTSC13* (Met/Cys +)	*dfg10Δ/dfg10Δ*
Fatty acid	Content of fatty acid (%)					
C14 Miristic	0,61±0,03	0,38 ±0,01	1,02 ±0,1	0,60 ±0,02	0,83 ±0,04	0,52 ±0,04
C16:0 Palmitic	19,83 ±0,08	18,03 ±0,28	17,08 ±0,52	17,35 ±0,12	18,09 ±0,17	16,93 ±0,58
C16:1 Palmitoleic	17,29 ±0,21	14,28 ±0,03	25,02± 1	15,05 ±0,21	18,21± 0,18	13,76± 0,66
C17:0 Margaric	0,46 ±0,34	0,36 ±0,04	0,55 ±0,02	0,75 ±0,01	1,29 ±0,03	0,46 ±0,04
C17:1 Margarinic	1,37 ±0,03	0,76 ±0,62	3,00 ±0,07	2,24 ±0,08	4,45 ±0,28	0,77 ±0,7
C18:0 Stearic	4,2± 0,08	4,04± 0,02	2,57± 0,13	3,73± 0,02	2,98 ± 0,24	4,05± 0,16
C18:1 Oleic	21,27± 0,13	17,44± 0,11	18,12 ± 0,33	19,86± 0,22	15,68± 0,23	16,66 ± 1,37
C18:2 Linoleic	26,52 ±0,4	34,43± 0,06	25,27 ±0,72	26,47± 0,05	26,30± 1,12	31,29± 1,03
C18:3 Linolenic	7,42 ± 0,09	7,88± 0,17	3,11± 0,22	7,26± 0,73	5,75± 0,42	7,62± 0,17

The values shown do not add to 100% because of a variable content of C17 fatty acids [between ca. 1 and 6% of all fatty acids analyzed, not shown].

Table S2 Primers used for mutant construction

**Name**	**Sequence**	**Remarks**
DFG10 F1F	GAGCTCTGGCAGCGGGTAGTTTAA	Restriction site for SacI underlined
DFG10 F1R	AGATCTCTACGTAAGTTTTGATGG	Restriction site for BglII underlined

DFG10 F2F	GGATCCGCTATGGCAAAAGAGCAA	Restriction site for BamHI underlined
DFG10 F2R	CTGCAGTCGCACTGTTATAGGGCA	Restriction site for PstI underlined
FverDFG10	CATAGTTTGTATGGACGTGTG	
RverDFG10	ATCTAGGCCATGTAGTAGCTG	
FverhisG	CGATACAGACCGGTTCAGAC	
RverhisG	GCGCTTTCAGTTTCTCCATG	
TSC13 F1F	GAGCTCCAGTTGCAAGAGAGATTC	Restriction site for SacI underlined
TSC13 F1R	AGATCTTCTTGAACTGACGAGTCC	Restriction site for BglII underlined
TSC13 F2F	GGATCCCTTTGTTGATCTTGTGGGT	Restriction site for BamHI underlined
TSC13F2R	CTGCAGCCAATACCCCAGCTTCAG	Restriction site for PstI underlined
FverTSC13	TCCCAATCTGAAGACATGATCGAT	
RverTSC13	TGTGTAGACTGGTATATCCCT	
TSC13MET-F	TTTGAGGAAATAATCCACAGTTGGTTAGCACACGAAAAGCAAGATTACAAGTAAAGATCTTTAGTGAGTGAATTGTCTATTCCAAGC	
TSC13TMET-R	TCAATTCATTGGAGGAAAAAGATTTAATGGATCTGGAACGTGATTTGACTTCAATGTCAGTCATAAGTGACAACACTTGGAAGTTGAA	

Table S3 Primers used for RT qPCR analysis

Symbol	Name	Reference sequence	Primers		Efficiency (%)
DFG10	*Candida albicans* SC5314 putative polyprenol reductase	XM_707500.2	F	AACCTCAAAGATGAACGTGAGC	2.32
			R	CTTAGCCCCAAATAGGTAGCC	
TSC13	*Candida albicans* SC5314 trans-2-enoyl-CoA reductase (NADPH)	XM_713023.2	F	TGTCGTGGTTAGCCTATGCTTT	2.23
			R	GCCACATTTGACCGGAACTAAC	
HMGR	*Candida albicans* SC5314 hydroxymethylglutaryl-CoA reductase (NADPH) (HMG1)	XM_708543.2	F	TGAACGTTTCTGTGTCAATGCC	2.21
			R	GTTGAGCATTTGCACCTGGATT	
ERG20	*Candida albicans* SC5314 bifunctional (2E,6E)-farnesyl diphosphate synthase/dimethylallyltransferase (ERG20)	XM_707824.2	F	TCCAGAACAAATTGGGAAAATCGG	2.35
			R	CGTTGTTCAGGGGTAGCAATCA	
ACC1	*Candida albicans* SC5314 putative acetyl-coenzyme-A carboxylases (ACC1)	XM_713531.1	F	AGGTGAAGAGCGTCATGTCATT	
			R	ATGTGGCACCAGCAATTAAACC	
RER2	*Candida albicans* SC5314 ditrans, polycis-polyprenyl diphosphate synthase (RER2)	XM_716843.1	F	GTTGTGTGAGGAGTATGGAGTT	2.32
			R	ACACGGCACGAGTATTGTT	
SRT1	*Candida albicans* SC5314 ditrans, polycis-polyprenyl diphosphate synthase (SRT1)	XM_019475163.1	F	CCCTATACTGCTCGTGATGAAAT	2.11
			R	ACGATCATGAAGTTCTCCTGAAA	
NUS1	*Candida albicans* SC5314 ditrans, polycis-polyprenyl diphosphate synthase	XM_718081.1	F	CACAAGTGATTAGGGAGGACATT	2.25
			R	GACCATGCTGTCAACTCACTAA	

ACT1	*Candida albicans* SC5314 actin (ACT1)	XM_019475182.1	F	TTGGATTCTGGTGATGGTGTTA	2.22
			R	TCAAGTCTCTACCAGCCAAATC	
CPA1	*Candida albicans* SC5314 carbamoyl-phosphate synthase (glutamine-hydrolyzing) (CPA1)	XM_717609.2	F	GGGAGAATTACGATTGGTGAAGA	2.25
			R	GGTGCCTTGGTGGTTACTTTA	
RPP2b	*Candida albicans* SC5314 ribosomal protein P2B (RPP2B)	XM_718047.1	F	TGGTAACACCTCTCCATCAGC	2.26
			R	GGATTCTTCGGCTTCAACACC	
RIP1	*Candida albicans* SC5314 ubiquinol-cytochrome-c reductase catalytic subunit (RIP1)	XM_019475318.1	F	GGTTCTGTCCATGTCACGGT	2.25
			R	GTTCAATGGAGCTGGACCCT	
LSC2	*Candida albicans* SC5314 succinate-CoA ligase (GDP-forming) subunit beta (LSC2)	XM_019475549.1	F	CACCACTCCAGAAGGTGCTT	2.23
			R	ACCACGACCACCAGTCAAAG	
PMA1	*Candida albicans* SC5314 H(+)-exporting P2-type ATPase (PMA1)	XM_712666.2	F	GCTGCCACAGTCAATGAAGC	2.20
			R	GCAATACCAACGGCATCACC	
